# Serum level and clinical significance of vitamin E in children with allergic rhinitis

**DOI:** 10.1186/s12887-020-02248-w

**Published:** 2020-07-31

**Authors:** Shi-yi Wang, Yin-feng Wang, Chun-chen Pan, Jing-wu Sun

**Affiliations:** 1 Department of Otorhinolaryngology-Head and Neck Surgery, The First Affiliated Hospital of University of Science and Technology of China, No. 17 Lujiang Road, Hefei, Anhui Province 230001 People’s Republic of China; 2 Department of Otolaryngology-Head and Neck Surgery, Anhui Provincial Hospital Affiliated Anhui Medical University, No. 17 Lujiang Road, Hefei, Anhui Province 230001 People’s Republic of China; 3 Department of Otolaryngology-Head and Neck Surgery, Affiliated Hospital of Yangzhou University, No. 368 Hanjiang Middle Road, Yangzhou, Jiangsu Province 225001 People’s Republic of China

**Keywords:** Allergic rhinitis, Vitamin E, Children, Skin prick test, Total IgE, Specific IgE

## Abstract

**Background:**

Allergic rhinitis (AR) is one of the most prevalent allergic diseases in children. This study aimed to investigate the association between serum concentrations of vitamin E and AR to determine if the vitamin E level is correlated with the occurrence and severity of AR.

**Methods:**

A total of 113 children were enrolled in this cross-sectional study. Sixty-five children in the outpatient group were diagnosed with AR, and 48 healthy children were recruited as controls. All subjects underwent serum vitamin E (adjusted for total cholesterol and triglycerides) measurements. Serum to total IgE (tIgE), the five most common allergen-specific IgE (sIgE) levels and skin prick test (SPT) were measured in children with AR. The severity of AR was assessed with the nasal symptoms score, and the situation of exposure to passive smoking were inquired.

**Results:**

Serum vitamin E levels were significantly lower in the AR group than in the normal children (*P* < 0.001). A significant negative correlation was observed between serum vitamin E levels and sIgE as well as the SPT grade. Serum vitamin E levels were also inversely related to the nasal symptoms score; however, statistical significance was not found.

**Conclusions:**

A significantly lower vitamin E level was found in children with AR. Lower serum vitamin E levels may have correlation with the occurrence of AR in children. However, serum vitamin E levels were not statistically correlated with the severity of AR.

## Background

Allergic rhinitis (AR) is widely defined as nasal mucosal inflammation and mostly reflects IgE-mediated type 1 hypersensitivity to common environmental and food antigens. Typical symptoms of the disease are nasal congestion, itching, sneezing and rhinorrhoea [1]. In patients with AR, elevated total IgE (tIgE) or specific IgE (sIgE) antibody levels are commonly observed. Thus, the serum levels of tIgE and sIgE after exposure to common allergens be used as a universal indicator for the diagnosis of AR and its severity [2].In recent years, the prevalence of AR and asthma is increasing worldwide, especially in children. The mechanism has not been fully elucidated hitherto, and increase in ambient PM_2.5_ levels and antioxidants vitamin D deficiency may be the main risks factors [3–5], while the association with antioxidants vitamin E deficiency is still unclear.

Vitamin E, a essential nutrient for reproduction, is synthesized in plant organisms and composed of eight fat-soluble compounds(α-, β-, γ-, δ-tocopherol, and α-, β-, γ-, δ-tocotrienol) [6].Some studies have confirmed that oxidative stress plays an important role in the pathogenesis of several allergic diseases, including AR [7]. Vitamin E is a peroxyl radical scavenger that can protect neurons and respiratory mucosa from oxidant damage, while vitamin E can significantly reduce the incidence of asthma and relieve respiratory mucosa inflammation [8, 9].

The relationship between dietary antioxidants and allergic diseases has been previously reported. Vitamin E intake can protect against the development of atopy and wheezing in young children [10]. However, vitamin E supplementation did not reduce the severity of AR or the duration that allergy medications were used to control symptoms in adults [11].

To date, there have been few researches on the association between AR and serum vitamin E in children. Many studies have focused on allergic diseases such as asthma, wheezing, and atopic dermatitis. Given that the potential therapeutic value of vitamin E in other allergic diseases, we addressed this deficiency by concentrating on the association between vitamin E and AR in children and determining the correlations between vitamin E levels and sIgE/tIgE, SPT grade and nasal symptoms score. The aim of this work was to evaluate the possible association between vitamin E levels and the occurrence and severity of AR in children.

## Methods

### Research Subjects

A total of 65 children aged from 6 to 14 years who were diagnosed with AR at the outpatient department of Anhui Provincial Hospital, Anhui, China, between September 2017 and September 2018 were included in the study. Forty-eight matched healthy children were recruited as controls at the same time. All studies are approved by Anhui Provincial Hospital Ethics Committee. The parents’ informed consent was obtained at enrolment. Sex, age, BMI and passive smoking were collected for all children. The diagnosis of AR was defined according to Allergic Rhinitis and Its Impact on Asthma guidelines [1].The inclusion criteria were 2 or more symptoms of nasal congestion, itching, sneezing and rhinorrhoea, totalling more than one hour a day; definite provocative factors; and skin prick test results positive for at least one allergen. All patients had symptoms without evidence of infection, sinusitis, otitis media, nasal polyps, nasal septum deviation, atopic dermatitis, lung diseases, systemic diseases and anatomical abnormalities. Within 3 months of the study, none used vitamin supplements, corticosteroids (nasal or systemic), anti-inflammatory drugs, anti-leukotrienes, cromolyn, or immunotherapy.

### Blood samples

Venous blood samples (2 ml) were obtained from children who had fasted for 8 h; the samples were then left to clot for 60 min at room temperature. After centrifugation for 15 min at 1500 rpm, serum samples were stored and frozen at − 80 °C until the day of the assay.

Serum levels of vitamin E were determined by HPLC (LC-20AD; Shimadzu, Japan) and were adjusted by total cholesterol and triglycerides (LabtestDiagnostica SA, Lagoa Santa, Brazil). All measurements were performed by the same researcher in the same laboratory and using the same kit.

Serum sIgE and tIgE were measured using ImmunoCAP (Phadia AB, Uppsala, Sweden) [12].Specific IgE concentrations were measured for the five most common allergens found in southern China (*Dermatophagoides farina*, cockroach, dandelion, ragweed, birch). The sIgE value of 0.35kU/L or above (0.35-100kU/L) was considered positive. sIgE were classified into 6 degrees [13]: grade 0: values < 0.35 kU/L, 1 grade: 0.35 ≤ values ≤ 0.69kU/L, 2 grade: 0.7 ≤ values ≤ 3.4kU/L, 3 grade: 3.5 ≤ values ≤ 17.4kU/L, 4 grade: 17.5 ≤ values ≤ 49.9kU/L, 5 grade: 50 ≤ values ≤ 100 kU/L, and 6 grade: values ≥ 100 kU/L.

### Symptoms Score

Children were instructed to complete the nasal symptom score questionnaire (maximum18). We scored the severity and duration of three nasal symptoms (nasal congestion, rhinorrhea and sneezing). The severity and duration of each nasal symptom were scored on a 3-point scale as follows: not at all/none of the time was 0; mild, well-tolerated/once in a while was 1; moderate, somewhat bothered/sometime was 2; and severe, very bothered/most or all time was 3.

### Skin prick testing

SPT was performed using standard allergen extracts (ALK Horsholm, Denmark) of 8 inhalant allergens (*Dermatophagoides farina*, *Dermatophagoides pteronyssinus*, *Blomia tropicalis*, birch, mugwort, ragweed, dandelion, cat hair, and cockroach). Histamine dihydrochloride (10 mg/ml) and physiological sodium chloride (9 mg/ml) served as positive and negative controls, respectively. SPT was performed on the volar surface of the forearm with a single peak lancet, and wheal sizes were measured after 15 min. Diameter of the wheal = 1/2 (longest diameter + short diameter), and the wheel diameter ≥ 3 mm is considered as positive [14]. The skin index (SI) was calculated as SI = allergen diameter/histamine diameter; values were recorded as normal: “˗“, negative; “+”, SI < 0.5; “++”, 0.5 ≤ SI < 1; “+++”, 1.0 ≤ SI < 2.0; “++++”, SI ≥ 2.0. If the reaction to the physiological sodium chloride prick was positive, skin irritation should be considered and eliminated. Those with a negative histamine reaction were also eliminated. The positive skin prick test response to *Dermatophagoidesfarina* (Df) was included in the correlation study.

### Statistical analysis

All statistical analyses were conducted using SPSS22.0 (SPSS, USA). Continuous variables were expressed as the mean ± standard deviation (SD) $\left(\bar{x}\pm \text{S}\right)$; differences between groups were determined by Student’s t-test or analysis of variance for continuous variables and by the Pearson chi-square test for categorical variables. Binary logistic regressions were designed to adjust the simultaneous effects of confounding variables such as age, sex, body mass index and exposed to passive smoking on the vitamin E level between the two groups. Correlations between serum vitamin E and sIgE, total IgE, the nasal symptoms score and SPT grade were calculated with multiple linear regression. A value of p < 0.05 was considered significant.

## Results

### Comparison of general characteristics and serum VE levels between AR children and control

**Fig. 1:**
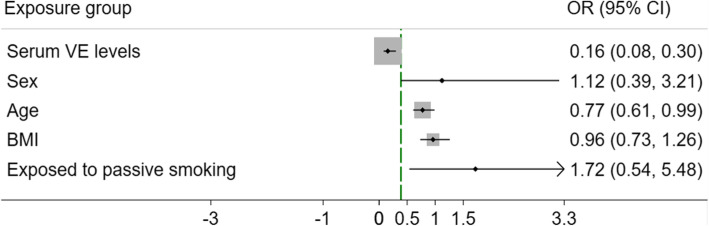
Risk of AR in children Odds ratios and 95% confidence intervals are presented to show the risk of AR among children exposed to different risk factors

Sixty-five children who were eventually diagnosed with AR were enrolled in the study, detailed in Table 1, and 48 healthy children were recruited as a control group in the same period. There were no significant differences regarding age, sex, body mass index or passive smoking between the two groups. The preliminary results revealed that serum vitamin E levels (ng/ml ± SD) were significantly lower in AR children (6.61 ± 1.37) than in normal children (9.21 ± 1.69; *P* < 0.001). Serum tIgE (IU/ml) was significantly higher in AR children (289.0 ± 101.1) than in normal children (82.5 ± 18.9; *P* < 0.001). Data are shown in Table 2. After adjusting the model for sex, age and body mass index, the analysis revealed that serum vitamin E (odds ratio [OR], 0.155; 95% confidence interval [CI], 0.08–0.3; *P* < 0.001) contributed significantly to AR, as shown in Fig. 1.

**Table 1:** General characteristics and clinical data of children with AR

	Serum VE levels (ng/ml)	Sex (M/F)	Age (yr)	BMI (kg/m^2^)	Passive smoking (Y/N)	tIgE (IU/ml)	sIgE grade	SPT grade	Allergen wheal diameter (mm)	Nasal Symptom score
1	10.52	M	< 8.3	14.33	N	203.6	0	+	3	4
2	9.66	M	8.3 ~ 11.5	18.43	N	426.9	2	++	6	10
3	9.43	F	> 11.5	17.66	N	436.9	1	+	3	14
4	9.16	M	8.3 ~ 11.5	17.98	Y	183.4	1	+++	4	8
5	8.99	F	> 11.5	16.78	Y	245.2	0	++	3.5	6
6	8.56	F	> 11.5	17.98	N	122.8	3	+	5	9
7	8.54	M	< 8.3	17.34	N	338.6	3	++	5	14
8	8.33	F	8.3 ~ 11.5	17.69	Y	269.2	3	+	3.5	11
9	8.23	M	< 8.3	16.89	N	279.5	3	++	6	16
10	8.14	F	< 8.3	18.67	N	357.9	1	++	4	10
11	8.04	M	< 8.3	16.45	N	124.2	3	++	4	15
12	8.03	F	> 11.5	19.11	N	163.1	1	˗	2	15
13	7.88	M	< 8.3	18.34	Y	219.1	2	+	3	6
14	7.81	M	> 11.5	18.54	N	326	1	+	3.5	16
15	7.68	M	< 8.3	21.32	N	206.2	2	++	5	8
16	7.34	M	< 8.3	16.44	Y	193.9	2	+	3.5	7
17	7.33	M	> 11.5	18.21	Y	199.8	3	+	4	12
18	7.31	M	8.3 ~ 11.5	17.89	Y	167.7	1	+	3	12
19	7.24	F	< 8.3	17.58	N	278.8	2	+	4	9
20	7.01	F	< 8.3	14.54	N	166.7	4	++	5	6
21	6.99	F	8.3 ~ 11.5	17.11	N	287.3	2	+++	10	5
22	6.97	M	< 8.3	16.46	Y	372.2	2	++	7	4
23	6.92	M	8.3 ~ 11.5	18.11	N	187.1	1	++	4	5
24	6.88	M	8.3 ~ 11.5	24.34	Y	380.5	4	+++	15	17
25	6.78	F	8.3 ~ 11.5	17.66	N	526.3	4	++++	15.5	16
26	6.73	M	8.3 ~ 11.5	18.98	N	269.3	4	−	0	8
27	6.71	M	> 11.5	25.29	N	443.7	4	++++	18	10
28	6.67	F	8.3 ~ 11.5	17.35	N	255.7	2	++	7.5	7
29	6.64	F	> 11.5	19.2	Y	416.1	2	++	5.5	9
30	6.55	F	> 11.5	15.33	N	428.1	1	++	6.5	15
31	6.55	M	8.3 ~ 11.5	18.46	N	359.9	2	−	0.5	8
32	6.53	F	8.3 ~ 11.5	17.77	N	221.8	4	+++	11	9
33	6.43	M	> 11.5	19.63	Y	297.1	1	++	6.5	13
34	6.43	F	> 11.5	18.84	Y	276.8	3	+++	12.5	17
35	6.33	F	8.3 ~ 11.5	17.9	N	228.2	3	−	0	13
36	6.22	M	< 8.3	16.5	N	316.5	3	+++	11	12
37	6.17	M	8.3 ~ 11.5	23.33	Y	179.3	2	++	7.5	6
38	6.15	F	8.3 ~ 11.5	17.76	N	218.7	4	++	4.5	7
39	6.08	M	< 8.3	16.03	Y	393.1	4	+++	10	10
40	6.05	M	> 11.5	19.34	N	293.3	4	−	0	13
41	6.02	F	8.3 ~ 11.5	15.11	N	290.4	3	+++	13	15
42	5.94	F	8.3 ~ 11.5	17.39	Y	387	1	−	2	7
43	5.9	M	8.3 ~ 11.5	19.44	Y	233.3	3	+++	10	16
44	5.86	F	> 11.5	18.76	N	510.4	1	++	7	11
45	5.83	M	> 11.5	18.45	N	139.5	2	++	5.5	10
46	5.76	M	8.3 ~ 11.5	18.54	Y	267.3	4	+++	9	15
47	5.76	M	> 11.5	17.98	N	294.4	4	++++	21	13
48	5.72	M	8.3 ~ 11.5	18.99	N	188.4	1	++	6	9
49	5.69	F	> 11.5	16.17	Y	266.1	4	+++	13	12
50	5.64	F	< 8.3	16.3	Y	335.8	2	++	7.5	11
51	5.53	F	> 11.5	16.87	N	165.6	5	+++	14	9
52	5.48	F	8.3 ~ 11.5	17.36	N	358.1	4	+++	11	18
53	5.42	M	8.3 ~ 11.5	17.8	N	278.2	3	++	5	6
54	5.33	F	< 8.3	17.89	Y	359.2	6	+++	13	13
55	5.21	M	> 11.5	19.23	Y	199.2	3	++	6	8
56	5.13	F	< 8.3	16.87	N	237.8	4	+++	13	11
57	5.08	F	< 8.3	15.05	N	207.6	3	+++	7.5	9
58	5.04	F	< 8.3	16.52	Y	286.3	4	++	7	11
59	5.02	M	> 11.5	17.91	N	284.8	6	++++	22.5	12
60	5.01	M	< 8.3	16.41	Y	243.7	4	+++	16	14
61	5.01	M	< 8.3	17.56	N	221.6	5	++++	19	10
62	4.92	F	8.3 ~ 11.5	18.29	N	389.1	4	+++	14	13
63	4.9	F	> 11.5	18.61	N	433.6	6	−	1	14
64	4.37	M	8.3 ~ 11.5	17.59	Y	578.1	5	++++	19.5	18
65	3.74	M	> 11.5	23.48	Y	367.5	6	++++	20.5	14

*yr* Year, *VE* Vitamin E, *AR* Allergic rhinitis, *M *Male, *F *Female, *BMI *Body mass index

**Table 2:** Clinical features and serum vitamin E levels in 2 study groups^a^

	AR (*n* = 65)	N (*n* = 48)	*P*-value
Sex(M/F)	35/30	26/22	0.973^b^
Age (yr)	10.0 ± 2.4	10.2 ± 2.3	0.868^c^
BMI(kg/m^2^)	18.03 ± 2.02	18.28 ± 1.81	0.506^c^
Serum total IgE (IU/ml)	289.0 ± 101.1	82.5 ± 18.9	< 0.001^c^
Serum VE levels (ng/ml)	6.61 ± 1.37	9.21 ± 1.69	< 0.001^c^
Exposed to passive smoking (%)	25(38)	11(23)	0.079^b^

*yr *Year, *VE *Vitamin E, *AR *Allergic rhinitis, *N *Normal, *BMI *Body mass index

^a^Values are presented as the mean ± SD or ratio

^b^Pearson chi-square test

^c^Student’s t test

### Correlative analysis of serum VE level and relevant indicators of AR

In the children with AR, there was a significant inverse correlation between serum vitamin E levels and sIgE (B=-0.577; *P* < 0.001). However, there was no significant inverse correlation between serum vitamin E and tIgE (B=˗0.002; *P* = 0.301), and there was no significant inverse correlation between serum vitamin E levels and the nasal symptoms score (B=-0.068; *P* = 0.160). The results of the skin prick test showed that 62 children were allergic to dermatophagoids, of whom 58 were allergic to *D.farina*, which was the most common allergen. There was a significant inverse correlation between serum vitamin E levels and Df SPT grade (B=-1.014; *P* < 0.001).Data are shown in Table 3.

**Table 3:** Multiple linear regression models for associated factors with VE in children with AR

	B	SE B	β	*P* value
**Model 1: tIgE and VE (*N *= 65)**				
Constant	8.739	1.862		< 0.001
tIgE	-0.002	0.002	-0.135	0.301
Sex	-0.162	0.379	-0.060	0.669
Age	-0.092	0.078	-0.166	0.244
BMI	-0.017	0.102	-0.025	0.869
Exposed to passive smoking	-0.366	0.355	-0.131	0.307
**Model 2: sIgE and VE (*N* = 65)**				
Constant	9.087	1.436		< 0.001
sIgE	-0.577	0.089	-0.630	< 0.001
Sex	-0.126	0.288	-0.046	0.663
Age	-0.105	0.060	-0.190	0.085
BMI	0.032	0.078	0.047	0.687
Exposed to passive smoking	-0.398	0.274	-0.143	0.151
**Model 3: SPT grade and VE (*N* = 58)**				
Constant	9.315	1.456		< 0.001
SPT grade	-1.038	0.154	-0.681	< 0.001
Sex	-0.189	0.305	-0.067	0.539
Age	-0.058	0.063	-0.102	0.358
BMI	0.043	0.079	0.072	0.546
Exposed to passive smoking	-0.627	0.289	-0.220	0.035
**Model 4: Nasal symptoms scores and VE (*N* = 65)**				
Constant	9.071	1.868		< 0.001
Nasal symptoms scores	-0.068	0.048	-0.182	0.160
Sex	-0.220	0.370	-0.081	0.555
Age	-0.074	0.079	-0.134	0.352
BMI	-0.029	0.100	-0.042	0.776
Exposed to passive smoking	-0.364	0.353	-0.131	0.306

R^2^ = 0.081 for model 1; R^2^ = 0.454 for model 2; R^2^ = 0.511 for model 3; R^2^ = 0.095 for model 4. *B *Unstandardized coefficient, *SE *Standard error, *β *Standardized coefficient;

## Discussion

We investigated the association between serum vitamin E and AR in children aged 6–14 years. The levels of vitamin E in children with AR were lower than normal children, and an association was found between vitamin E levels and AR. The results remained significant even after adjusting for confounding factors related to vitamin E.

The relationship between AR prevalence and serum vitamin E levels is controversial in existing studies. In several cohort studies, it was found that maternal vitamin E intake from food during pregnancy was inversely related to the risk of AR in children [15, 16]. Similarly, high-dose vitamin E supplementation in combination with routine treatment may be valuable to improving symptoms in patients with seasonal allergic rhinitis [11]. In contrast, some reports found no association between AR and vitamin E intake [17, 18]. The differences may derive from the following reasons: (1) Children may need more supplementation in the vitamin E due to their faster metabolic rate (2) the discrepancy in dietary structure and nutritional status of different regions.

Obtaining a thorough history and physical examination as well as identifying specific allergic triggers are required to establish the clinical diagnosis of allergic rhinitis. Allergen-specific IgE tests and skin prick tests are the main methods for determining allergens. Each has its advantages and cannot be replaced by the other. Therefore, we analyzed the correlation between the two results and serum vitamin E levels of children with AR, attempting to link vitamin E with the diagnosis of AR. In this study, 62 children with AR were found to be allergic to dermatophagoids. *D.farina*is, the most common allergen in children with AR, followed by cockroach and birch. At the same time, our results showed a significant inverse correlation between serum vitamin E levels and Df SPT grade.

McCann W A et al. [19] demonstrated the diagnostic value of serum sIgE in allergic diseases, with the sensitivity fluctuating between 84% and 95% and the specificity fluctuating between 85% and 94%. Given that serum immunoglobulin E (IgE) can be synthesized even before clinical symptoms occur, and elevated IgE levels are one of the indicators of type I allergic reactions, we measured the serum tIgE and sIgE levels and found that’s IgE was negatively correlated with vitamin E. Correspondingly, Fogarty et al. [20]revealed that higher-dose of vitamin E intake were associated with lower serum IgE concentrations and a lower frequency of allergic sensitization. Because of the diagnostic value of SPT and sIgE in AR, we investigated the association between SPT and sIgE and serum vitamin E and found a statistically significant inverse correlation, which may reflect the inverse correlation between serum vitamin E level and AR. However, the correlation between tIgE and vitamin E was not statistically significant. This may be because many factors can lead to increasing tIgE levels in the body, and approximately one-third of patients with AR were in the normal range.

Vitamin E, a fat-soluble vitamin, is one of the most important antioxidants and is closely correlated with immune function [21].At the same time, the production of reactive oxygen species (ROS) and oxidative stress are related to allergic inflammation. Vitamin E metabolites have been identified as potential regulator associated with immune, inflammatory responses, lipid metabolism, neuronal cell protection and vessel homeostasis in vivo [22, 23]. Passive smoke exposure has been reported to exacerbate NOX2 activation and higher oxidative stress levels in children with AR [24]. We compared the exposure of children exposed to passive smoking between the two groups, and found that compared with the control group, there were more exposed children, but the difference was not statistically significant, which may be related to the level and the type of passive smoking exposition [25, 26]. A population-based study demonstrates that exposure to passive smoking predominantly effects non-allergic rhinitis and more pronounced in adolescents, rather than allergic rhinitis [27]. Although vitamin E has a positive antioxidant effect and oxidative stress has a role in the development of AR, the association between vitamin E and AR has not been proven beyond any reasonable doubt. Our data provide solid evidence for the link between vitamin E and allergic rhinitis. T helper 2 (Th2) cytokinesinterleukin-4 (IL-4), IL-5, IL-9 and IL-13 play key roles in AR propagation and in the maintenance of allergic inflammation, while T helper 1 (Th1) cytokines play the opposite role. In vitro, vitamin E promotes the secretion ofTh1cytokine and inhibits Th2cytokines secretion [28, 29]. Vitamin E also reduces the secretion ofIL-4in human peripheral blood T cells [30]. IL-4 can promote the production of IgE antibodies by B-cells and is one of the key cytokines in the development of allergic inflammation. In addition, vitamin E inhibits the activity of cyclooxygenase, indirectly inhibits the synthesis of arachidonic acid-derived prostaglandin E_2_ (PGE_2_), and PGE_2_ has been involved in shifting the balance of Th1/Th2 cells and their cytokines towards a Th2 profile [31, 32]. In conclusion, these findings support that vitamin E might be protective against allergic sensitization.

Our study showed no significant inverse correlation between serum vitamin E levels and nasal symptoms scores. In a controlled study, 112 patients with seasonal AR had lower nasal symptoms scores after high-dose vitamin E supplementation (800 IU/d) during the hay fever season [11]. In contrast, another study showed that no significant effect on nasal symptoms in 63 patients with perennial AR after normal-dose vitamin E supplementation (400 IU/d) [18].The differences may be due to the subjectivity of the symptoms score and the fact that it is targeted at children; a larger sample and further clinical studies are needed to evaluate this relationship.

## Conclusions

Our study is one of a few to investigate the relationship between serum vitamin E and AR in children, and it is the first to explore the potential association of IgE and SPT. Our study has multiple strengths, including complete data on both serum vitamin E, IgE and SPT results of children with AR. Serum vitamin E, IgE and SPT results were also measured by trained health professionals.

We recognize that there are some limitations in this study. First, this study failed to detect markers of oxidative stress, missing a possible mediator between vitamin E and the occurrence of AR. Second, the severity of nasal symptom in AR was based on subjective assessments. Third, we utilized sIgE measurements from a single allergen, which may lead to the problem of partial generalization. Moreover, since our study was cross-sectional design, causality cannot be inferred. Further studies are needed based on prospective, longitudinal, and objective methods (e.g. anterior active rhinomanometry [33]) to confirm and expand our results.

In view of the significantly serum lower vitamin E levels in children with AR, we speculated that lower serum vitamin E levels may have correlation with the occurrence of AR in children, providing an objective basis for designing feasible intervention programs for children with AR. However, serum vitamin E levels were not statistically correlated with the severity of AR, requiring us to collect more samples in the future for prospective study to reveal the cause.

## Data Availability

The datasets generated during the study are not publicly available due to the risk of identifying participants but are available upon reasonable request.

## Abbreviations

AR: Allergic rhinitis
VE: Vitamin E
IgE: Immunoglobulin E
tIgE: Total IgE
sIgE: Specific IgE
SPT: Skin prick test
SI: Skin index
Df: Dermatophagoides farina
Th1: T helper 1
Th2: T helper 2
IL-4: Interleukin-4
PGE_2_: Prostaglandin E_2_
yr: Year
N: Normal
BMI: Body mass index
